# Non-Listening and Self Centered Leadership – Relationships to Socioeconomic Conditions and Employee Mental Health

**DOI:** 10.1371/journal.pone.0044119

**Published:** 2012-09-24

**Authors:** Töres Theorell, Anna Nyberg, Constanze Leineweber, Linda L. Magnusson Hanson, Gabriel Oxenstierna, Hugo Westerlund

**Affiliations:** Stress Research Institute, Stockholm University, Stockholm, Sweden; The University of Queensland, Australia

## Abstract

**Background:**

The way in which leadership is experienced in different socioeconomic strata is of interest per se, as well as how it relates to employee mental health.

**Methods:**

Three waves of SLOSH (Swedish Longitudinal Occupational Survey of Health, a questionnaire survey on a sample of the Swedish working population) were used, 2006, 2008 and 2010 (n = 5141). The leadership variables were: “*Non-listening leadership*” (one question: “Does your manager listen to you?” - four response categories), “*Self centered leadership*” (sum of three five-graded questions – “non-participating”, “asocial” and “loner”). The socioeconomic factors were education and income. *Emotional exhaustion* and *depressive symptoms* were used as indicators of mental health.

**Results:**

Non-listening leadership was associated with low income and low education whereas self-centered leadership showed a weaker relationship with education and no association at all with income. Both leadership variables were significantly associated with emotional exhaustion and depressive symptoms. “Self centered” as well as “non-listening” leadership in 2006 significantly predicted employee depressive symptoms in 2008 after adjustment for demographic variables. These predictions became non-significant when adjustment was made for job conditions (demands and decision latitude) in the “non-listening” leadership analyses, whereas predictions of depressive symptoms remained significant after these adjustments in the “self-centered leadership” analyses.

**Conclusions:**

Our results show that the leadership variables are associated with socioeconomic status and employee mental health. “Non-listening” scores were more sensitive to societal change and more strongly related to socioeconomic factors and job conditions than “self-centered” scores.

## Introduction

The way in which leadership is experienced in different socioeconomic strata is of interest in its own right. Differences in perceived leadership between different socioeconomic groups could indicate differences in employee perception but they could also reflect “real” differences in managerial practices towards employees in different socioeconomic strata. There is a well-documented relationship between poor socioeconomic status and poor working conditions [1]–[2]. A recent cross-sectional study [3] of hotel employees in Italy, Sweden and Poland showed that hotel managers in Italy were reported by their subordinates to be more autocratic and to show less integrity than in Poland and Sweden. Interestingly, employees in Italy also more often expected managers to behave in this way. In addition, a highly significant relationship between such negative perceived management styles and poor self-reported employee health was shown in that study. This illustrates that different “culturally” constructed management styles may develop in different cultures or groups. Such processes are likely also to arise differently in different social strata in the national context as well as in international comparisons. For example, it has been shown that Swedish leadership ideals or expectations on leaders differ markedly when comparing political leadership with leadership in the private sector [4]. Also, it has been concluded from a meta-analysis (of predominantly North American studies) that transformational leadership (a dimension, which includes individual consideration for employees) is reported to be more frequent on lower levels in organizations, and within the public sector [5]. This result could reflect what kind of behaviors that employees wish from their leaders on different organizational levels. Employees with lower possibilities to influence their work situation could be assumed to search for more social support from co-workers and managers in order to maintain their self-esteem, whereas professionals with more control may be satisfied with less support [6]. In accordance with the theory of power as a mediator in the work stressor-strain relationship is a finding indicating that the negative effect of abusive supervision was buffered when the individual was employed in a high-power customer service occupation [7]. Our focus in the present study is on social stratification of perceived management style in Sweden. Social class is an important determinant of health. This has been extensively reported in national as well as international studies [1]–[2]. There is also scientific evidence for a relationship between leadership and employee health. For instance, a recent prospective study from our group [8] showed that poor leadership - as it is perceived by employees - is associated with near-future risk of developing cardiovascular disease among subjects without previous such disease, even after adjustment for accepted cardiovascular risk factors such as age, smoking habits, body mass index, serum lipids, plasma fibrinogen and regular physical activity. The aim of the present study is to contribute to the understanding of the relationship between socioeconomic conditions and perceived leadership in relation to health.

On the basis of international studies of leadership styles we decided to focus on two dimensions of perceived leadership, one positive aspect, the degree to which the leader is perceived as listening (we will be using the opposite concept non-listening manager below) and one negative aspect, the degree to which the leader behaves in a self-centered way (self-centered manager). This dimension is conceptually close to a passive-avoidant leadership, associated with increased burnout rates among employees [9]. These two aspects were studied with hypotheses formulated in advance:

1. Perceived “listening manager” style and “self centered manager” style, respectively, are partly “explained” statistically by the employee's gender, income and education. Possible observed relationships between these socioeconomic factors and leadership ratings will vary over time (observations on three occasions with two-year intervals).
2. The prevalence of the two manager behavior styles will vary over time. Changes in perceived managerial styles will to some extent be related to changes in working life.

This study has been approved by the Regional Research Ethics Committee, Stockholm, Ref.no: 2006/158-31.

## Methods

### Study samples

**Figure 1:**
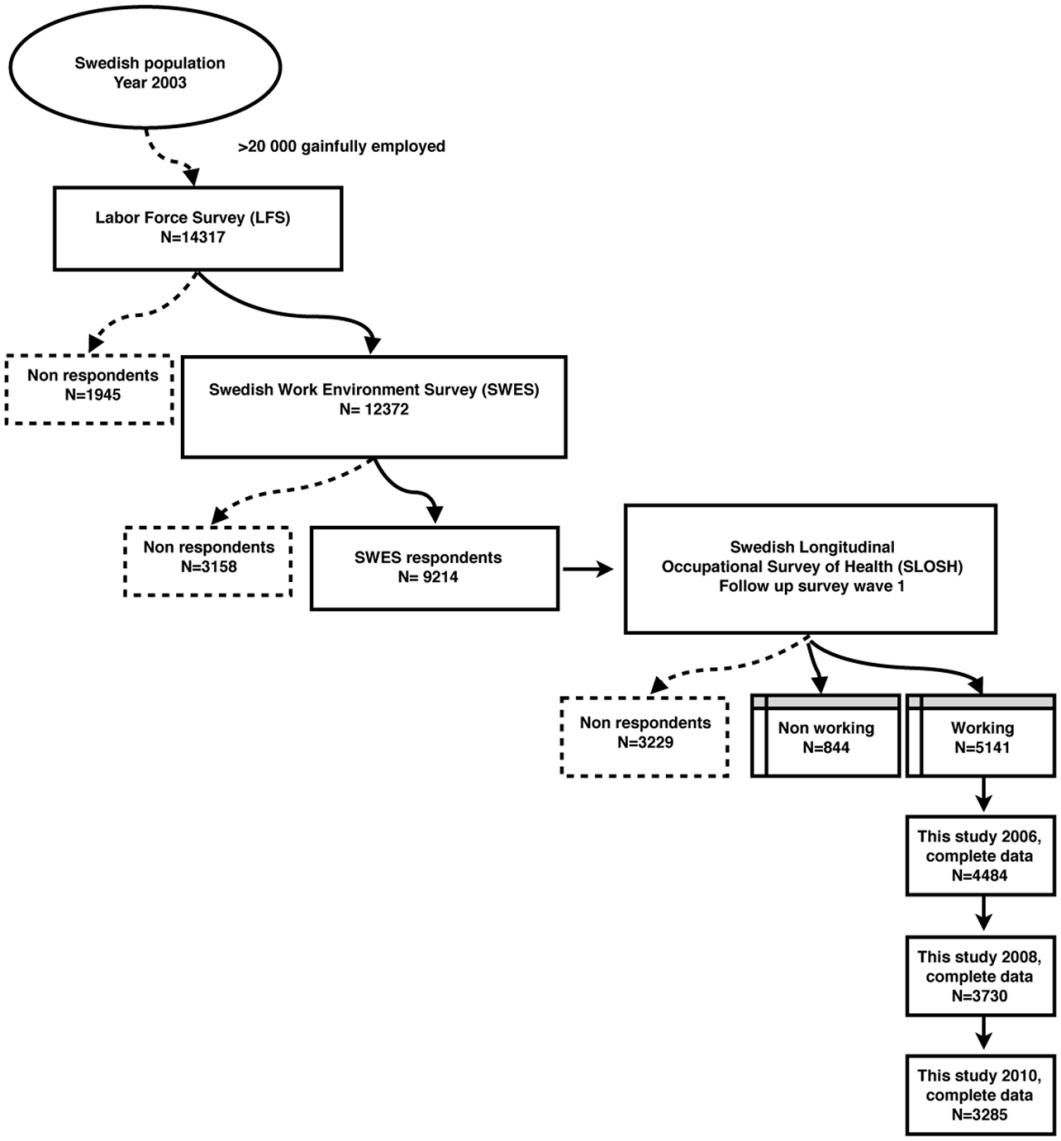
Flowchart showing recruitment and participation in 2006.

Figure 1 shows how the study cohort in 2006 was created. The SLOSH was originally recruited from the Swedish Work Environment Survey (SWES) which is conducted biennially by Statistics Sweden (SCB) and consists of subsamples of gainfully employed people, aged 16–64 years, from the Labor Force Survey (LFS, upper left square in the figure). These individuals had been sampled into the LFS through stratification by county of birth, sex, citizenship, and inferred employment status. This stratified random sample represents the full population of working Swedish men and women. The total participation rate in this first sampling step (from the general population to LFS) is estimated to 74%. Participants in LFS were then invited to participate in SWES. In this step (from LFS to SWES) the participation rate was 86%. Three years later eligible respondents to SWES 2003 (9154) were invited to enroll in the SLOSH (10) which was initiated by the Stress Research Institute at the Stockholm University in collaboration with Statistics Sweden in April 2006. The participation rate in this step was 65%.In the prospective part of the present study the participants in 2006 were followed up in 2008 and then again in 2010. Out of the 5141 working respondents in SLOSH in 2006, 4,484 respondents had complete data for the correlation analysis (87%) this year and in 2008 3269–3730 out of those 5141 respondents (see table 3) had complete data for statistical multivariate analyses (64%–73%). 6,5% of the participants in 2006 had reached retirement age until 2008. In the follow-up in 2010 (not shown) the numbers of participants in the analyses with complete data for multivariate analyses ranged from 2701 to 3285 (53%–64%). An additional 6,8% of the participants in 2006 had reached retirement age from 2008 to 2010. Two reminders by mail were used for minimization of drop-out. In 2008 a third reminder was sent in August. This resulted in a small addition of respondents (5%) which is included in the participation rates described above.

More detailed information about the cohort, response rate and characteristics of responders vs. non-responders has been published elsewhere [10]–[13]. There was no difference between responders and non-responders with regard to country of birth and citizenship.

### Assessments

The socioeconomic factors were education (five-graded scale, self-reported in the questionnaire, high score corresponds to high education) and yearly income (in hundreds of Swedish crowns per year, from tax registry; because the distribution was markedly skewed with a right sided tail, e log transformation was used, range 0–8,23). Age and gender were also included as explanatory variables.

The two selected leadership dimensions were:

Does your manager listen to you and pay attention to what you say? In this one-item “Non-listening leadership” variable there were four response categories ranging from “to a very high extent” to “a very small extent or not at all”. High score indicates poor condition (non-listening). Range 1–4 with mean 2,15 to 2,20 (2006, 2008 and 2010) and standard deviation 0,7–0,8.

“Self centered leadership”. This factor was calculated from three questions (non-participating, asocial and loner) according to results from factor analysis [13]. For each question there were five response categories ranging from “very infrequently” to “very often”. The items were added up to a sum score (range 3–15) with mean 5,9 (same for all study years) and standard deviation 2,5–2,6.

The two leadership variables were approximately normally distributed although the self-centered score was slightly skewed.

The effect of adjustment for part time work was tested for both variables but this did not have any statistical influence on the findings.

Two indicators of mental health were used:

1. Symptoms of emotional exhaustion, the most widely used of the three scales in the Maslach “Burnout” Inventory [14]. Range 5–30. Mean 10,76–11,98 with standard deviation 5,74–6,02.
2. Depressive symptoms according to a 6 item subscale of Hopkins Symptom Checklist (SCL) Depressions scale. [15]–[16]. This has been established as a subscale of the total SCL and has been utilized in Danish and Swedish population studies. It has six five-graded questions (total range 0–24). The distribution is slightly skewed. Mean 5,37–5,78 with standard deviation 5,12–5,30.

Psychological demands and decision latitude were measured by means of the Swedish shortened version (DCQ) [17] of the Job Content Questionnaire [1], [18] and were also used as explanatory variables. The demand score comprises five (total sum ranging from 5 to 20 with mean 12,88–13,33 and standard deviation 2,61–2,70) and the decision latitude score six (range 6 to 24 with mean 18,44–18,60 and standard deviation 2,72–2,89) four-graded questions. Both dimensions are close to normally distributed.

### Statistical methods

Since all the leadership and outcome variables as well as the work environment variables were close to normally distributed (skewness for none of them exceeding 1,0; the variable annual income in Swedish crowns was logarithmically transformed), statistics assuming normal distributions were used. First of all, product moment correlations were computed between all the study variables. Secondly, since the relationship between education and the leadership dimensions was crucial, the mean score of each one of the two leadership variables for each one of the five education levels was computed. In the final analyses statistical predictions of the three different health outcomes (from 2006 to 2008 and from 2006 to 2010 separately) were made by means of multiple linear regressions, with separate analyses for emotional exhaustion and depressive symptoms and for self centered and non-listening leadership respectively. These analyses were performed in two steps: 1) using gender, age, education and income as explanatory variables and 2) as in step 1, but with the addition of psychological demands and decision latitude at work. Since the relationship between education and leadership was crucial for this work, only the statistical contributions of education and leadership will be shown in the tables.

The leadership and health outcome variables were subjected to a one-way analysis of variance exploring changes over time.

## Results

Table 1 shows the correlations between all study variables in 2006. There was a high correlation (0,64) between the two health outcome variables emotional exhaustion and depressive symptoms. These two variables were treated separately since we felt that they reflected different aspects of mental health but were too close to one another statistically to be treated in the same equations. The two manager variables were also fairly strongly correlated (0,43) and were treated in different equations for the same reason. Income (e log transformed), education and decision latitude were positively correlated (r = 0,16–0,32). High decision latitude and high income were correlated with low emotional exhaustion and low depressive symptoms scores (r = −0,05–−0,13) whereas a high educational level showed small but positive significant correlations with the mental health indicators (0,04 for both). Psychological demands had strong correlations with emotional exhaustion (0,43) and depressive symptoms (0,27) and there were also significant correlations between decision latitude and emotional exhaustion (−0,11) and between decision latitude and depressive symptoms (−0,13).

**Table 1:** Product moment correlations between all study variables in 2006 (n = 4484).

	Age	Gender	Income (elog)	Education	Demands
Age	x				
Gender	,00	x			
Income(elog)	,28	'-,23	x		
Education	'-,20	,12	,16	x	
Demands	'-,04	,03	,11	,17	x
Dec. lat.	,07	'-.,06	,24	,32	.,07
Self centered	,01	'-,08	,00	'-,04	.,18
Non-listen	,01	,02	'-,06	'-,09	.,19
Emot. Exh.	'-,01	,11	'-,05	,04	.,43
Depressive	'-,08	,14	'-,08	,04	.,27

	Dec. lat.	Self centered	Non-listen.	Emot. Exh.	Depressive
Age					
Gender					
Income(elog)					
Education					
Demands					
Dec. lat.	x				
Self centered	'-.,12	x			
Non-listen	'-.,29	.,43	x		
Emot. Exh.	'-.,11	,21	,28	x	
Depressive	'-,13	,19	,24	,64	x

“Non-listening” leadership style showed modest and significant correlations with emotional exhaustion (0,28) and depression (0,24). The corresponding correlations between self centered leadership and the mental health indicators were slightly lower (0,21 for emotional exhaustion and 0,19 for depressive symptoms). “Self centered” leadership had no correlation at all with income (0,00) and a low but significant correlation with education (−0,04) whereas non-listening leadership showed small but more clear relationships with these socio-economic variables (−0,06 for income and −0,09 for education).

“Non-listening” leadership showed a small but significant variation over time (means 2,15, 2,14 and 2,24 for the years 2006, 2008 and 2010 respectively, F = 17,87, df = 2/2342, p = 0,0001). A similar tendency albeit less pronounced was found also for self centered leadership (means 5,83, 5,84 and 5,99, F = 4,29, df = 2/2258, p = 0,01). In addition the self-centered leadership variable was divided into its three parts with regard to variation over time. The “non-participating” item did indeed show significant variation over time with the worst mean score in the high unemployment third year 2010 (means 2,24, 2,20 and 2,35 with F time = 16,08 df = 2/2311 and p<0.0001) whereas the two other items, “asocial” and “loner”, did not show any significant variation over time (p = 0,572 for asocial and p = 0,282 for loner).

There was a small but significant between-year variation in psychological demands with a clear decrease in psychological demands in 2010 (M = 12,88 in 2010 versus M = 13,27 2006 and M =  13,33 2008 (F = 52,82, df = 2/2472, p = 0,0001). Decision latitude had its highest mean in the mid year 2008 (M = 18,63 2006, M = 18,79 2008 and M = 18,56 2010, F = 17,52,df = 2/2606, p = 0,0001).

**Figure 2:**
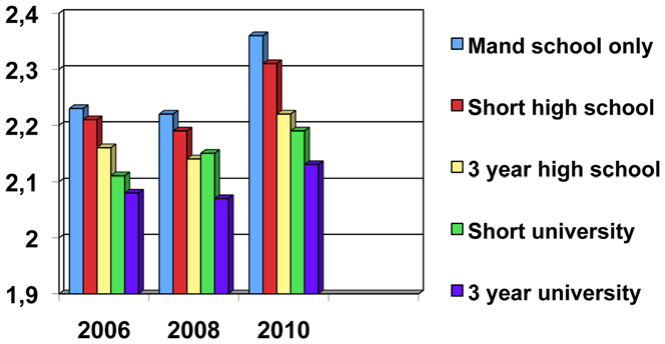
Mean scores in 2006, 2008 and 2010 for “non-listening” leadership in different educational strata. **P-values (association between education and ”non-listening” for all the three years p<0.0001.**

**Figure 3:**
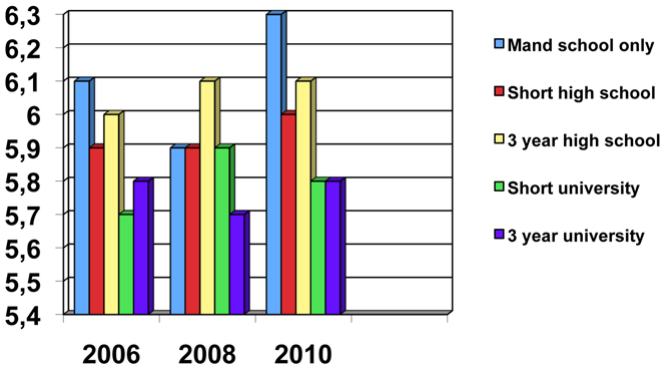
Mean scores in 2006, 2008 and 2010 for “self centered” leadership in different educational strata. **P-values (association between education and ”self-centered”) for 2006 0.013, for 2008 0.059 and for 2010 0.001.**

The mean leadership scores in different educational strata in the three study years are displayed in Table 2, Figure 2 and 3. The relationship between education and “non-listening” leadership is consistent and highly significant (albeit of small magnitude) for all the three study years whereas the relationship between education and “self centered” leadership is less consistent (non-significant for the year 2008) and also not as highly significant. Figure 2 also shows the increase in mean scores in “non-listening” leadership in the year 2010.

**Table 2:** Mean leadership scores in different educational strata and in the three study years.

	2006	2008	2010
***Self-centered leadership (3–15)***			
Mandatory school only	6,1 (2,7)	5,9 (2,5)	6.3 (2,5)
Number of subjects	899	539	403
Short high school education	5,9 (2,5)	5,9 (2,5)	6,0 (2,5)
Number of subjects	1061	786	629
At least 3 year high school	6,0 (2,7)	6,1 (2,8)	6,1 (2,6)
Number of subjects	964	673	589
Short university education	5,7 (2,4)	5,9 (2,6)	5,8 (2,4)
Number of subjects	682	499	425
At least 3 years university	5,8 (2,5)	5,7 (2,5)	5,8 (2,3)
Number of subjects	1227	941	812
***Non-listening leader (1–4)***			
Mandatory school only	2,23 (0,82)	2,22 (0,74)	2,36 (0,85)
Number of subjects	927	554	413
Short high school education	2,21 (0,79)	2,19 (0,72)	2,31 (0,87)
Number of subjects	1074	794	649
At least 3 year high school	2,16 (0,76)	2,14 (0,72)	2,22 (0,85)
Number of subjects	973	681	592
Short university education	2,11 (0,74)	2,15 (0,74)	2,19 (0,82)
Number of subjects	694	503	430
At least 3 years university	2,08 (0,72)	2,07 (0,72)	2,13 (0,82)
**Number of subjects**	**1243**	**956**	**829**

Finally a series of multiple linear regressions (Table 3) were computed in which the two indicators of health status (either for mental exhaustion or for depressive mood) along with rating of leader (“self centered” and “non-listening” in separate analyses), all of those in 2006, were used for prediction of the same health status variables in 2008 and in 2010. In these analyses a second analytical step was also taken in which psychological demands and decision latitude from 2006 were added to the predictors. These analyses (Table 3) showed that “self centered” leadership significantly predicted depressive mood (p = 0,004) in 2008 when adjustments had been made for sociodemographic variables and depressive mood in 2006. “Self centered” leadership was still a significant predictor when psychological demands and decision latitude in 2006 had been added to the equation although with reduced strength (p = 0.041). In a similar way it was possible to predict depressive mood in 2008 (p = 0,026) by means of the “non-listening” leadership score in 2006 even after adjustment for depressive score in 2006 as well as the demographic variables. The latter prediction however became non-significant after psychological demands and decision latitude had been taken into account (p = 0,334). All corresponding leadership based predictions of mental exhaustion in 2008 failed to reach statistical significance, and the leadership variables had no significant contribution in the four-year predictions (2006–2010).

**Table 3:** Education and leadership variables in 2006 as predictors of mental health scores (emotional exhaustion and depressive symptoms) in 2008.

*1a.) Emotional exhaustion without inclusion of psychological demands (PD) and decision latitude (DL) at work*	
n = 3673, self centered analysis	
Education	0,638+/−0,222, p = 0,004
Self centered	0,048+/−0,070, p = 0,473
n = 3730, non-listening analysis	
Education	0,644+/−0,219, p = 0,004
Non-listening	0,283+/−0,770, p = 0,716
*1b.) Emotional exhaustion with inclusion of psychological demands (PD) and decision latitude (DL) at work*	
n = 3556, self centered analysis	
Education	0,464+/−0,238, p = 0,051
Self centered	0,002+/−0,060, p = 0,961
n = 3614, non-listening analysis	
Education	0,500+/−0,236, p = 0,035
Non-listening	−0,427+/−0,807, p = 0,594
*2a.) Depressive symptoms without inclusion of PD and DL/work*	
*n = 3323, self centered analysis*	
Education	0,500+/−0,203, p = 0,014
Self centered	0,179+/−0,061, p = 0,004
n = 3378, non-listening analysis	
Education	0,458+/−0,202, p = 0,023
Non-listening	1,573+/−0,704, p = 0,026
*2b.) Depressive symptoms with inclusion of PD and DL/work*	
n = 3220, self centered analysis	
Education	0,405+/−0,219, p = 0,064
Self centered	0,132+/−0,064, p = 0,041
n = 3269, non listening analysis	
Education	0,368+/−0,216, p = 0,090
Non-listening	0,715+/−0,742, p = 0,334

Relative standardized linear beta coefficients +/−standard error of mean. Results from multiple linear regressions.

Variables included in the equations but not shown in the table were

a.) Age, gender, income (e log transformed SEK/year), emotional exhaustion score (1) and depressive symptom score (2) in 2006.

b.) as a.) but in addition psychological demands (PD) and decision latitude at work (DL/work).

Education was significantly (p = 0,004–0,035) or almost significantly (p = 0,051–0,090) and independently predictive in the positive direction (the higher the educational level the greater likelihood of a high depressive score). In addition (not shown in the table) education was significantly and independently predictive of emotional exhaustion score in the four-year predictions (2006–2010) after adjustments for emotional exhaustion in 2006 and socio-demographic variables as well as after the additional adjustments for psychological demands and decision latitude (p = 0,004–0,050). Educational level had no statistically significant independent value in the four-year predictions of depressive symptoms, however.

High psychological demands were independently and significantly predictive of both health outcomes both from 2006 to 2008 and from 2006 to 2010 in these equations. High decision latitude predicted significantly and independently low emotional exhaustion score in 2010 (with adjustment both for non-listening and self centered leader score).

**Figure 4:**
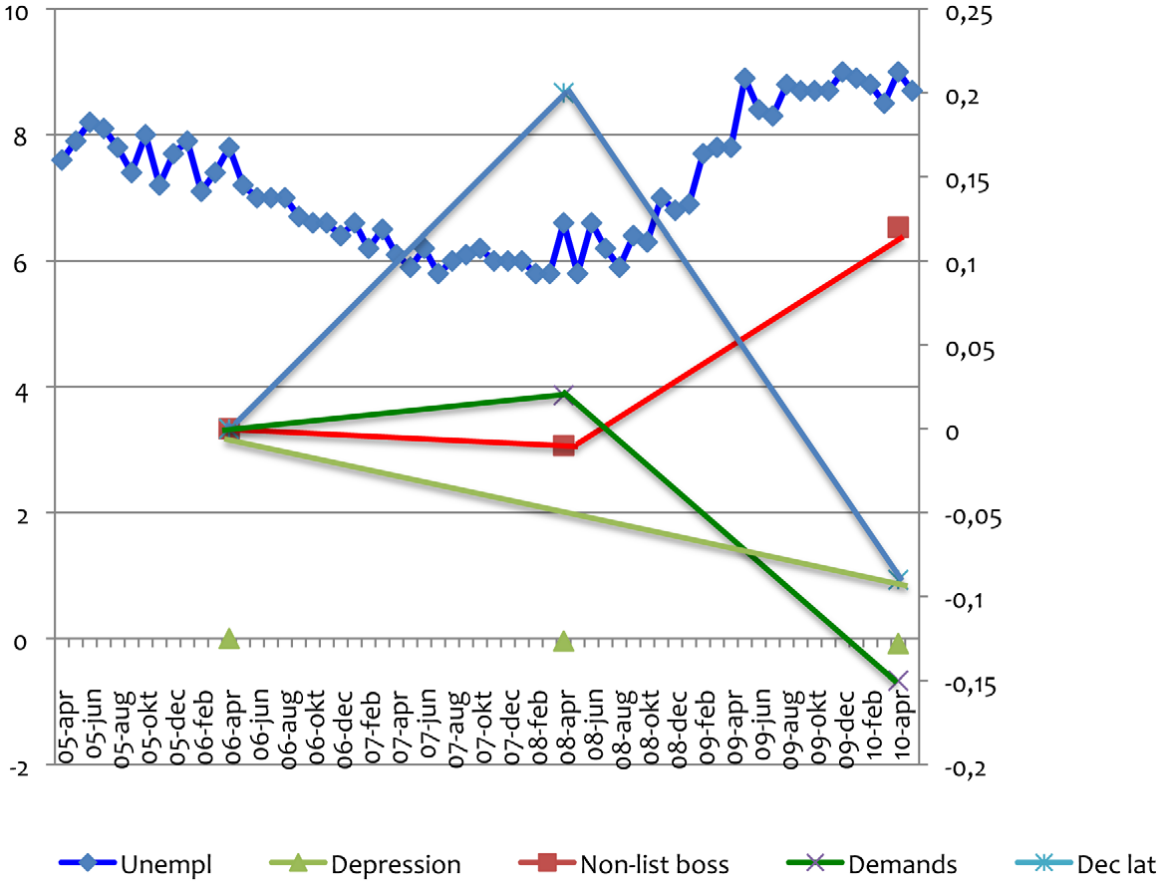
Relationship between unemployment rates (% unemployed 15–74 years of age from April 2006 to April 2010, y axis to the left) in Sweden according to official statistics. The vast majority of questionnaires in SLOSH were collected in April-May 2006, 2008, 2010. The diagram also shows changes in SLOSH means for non-listening manager (red), depressive symptoms (yellow), psychological demands (green) and decision latitude (blue) at work. Scores have been transformed to number of standard deviations, and 2006 is reference ( = 0). These numbers are displayed on the y axis to the right.

Figure 4 displays the relationship between unemployment rates (% unemployed 15–74 years of age from April 2006 to April 2010) in Sweden according to official statistics (upper part of diagram). The vast majority of questionnaires in SLOSH were collected in April-May 2006, 2008, 2010. The lower part of the diagram shows changes in SLOSH means for non-listening manager (red), depressive symptoms (yellow), psychological demands (green) and decision latitude (blue) at work. Scores have been transformed to number of standard deviations, and 2006 is reference ( = 0). Although the changes are small in absolute terms, the diagram illustrates lowered demands and increased non-listening leadership during the year with high unemployment (2010) as well as the fact that depressive symptom scores decreased continuously and that the decision latitude score was higher during the “good” year 2008 than during the other years.

## Discussion

The results showed that low education predicted both non-listening and self-centered leader scores. In addition low income was associated with a high non-listening leader score. Gender was related to the “self centered” leadership score; women described their managers as less self centered than did men. Age had no relationship with leadership scores.

Sweden experienced a financial crisis after April 2008. The unemployment rate rose from approximately 6% when our data was collected in April 2008 until 8.5% in April 2010. Management style, particularly “non-listening”, deteriorated this year. The changes in scores related to Swedish unemployment statistics – more “non-listening” managers, lower demands and fewer depressive symptoms during high unemployment - are not easy to explain. The continuous slight decrease in depressive symptoms from 2006 to 2010 could be age-related since high depressive symptom scores are significantly related to low age (table 1). The fact that the participants in this prospective part of our analyses (2006–2010) became four years older could give rise to a decrease in 0.04 standard deviations in depressive symptom score. The observed change was larger, 0.08 standard deviations, however, so ageing could not explain the whole tendency. For the other three variables displayed in Figure 4, age is not a likely explanatory factor at all. Perhaps a short downturn in the business cycle makes the managers insecure (and non-listening) while the tempo in the workplaces slows down. The fact that the reported employee decision latitude was high after a period of well-functioning business (2008) is an expected finding [1], [19], [20].

It should be cautioned that most of the observed associations have a small magnitude. This is true both in the analyses of changes over time and in the multiple regressions in which work environment variables are used for statistical explanation of health outcomes prospectively. Despite their small magnitude, however, they are potentially important on a population basis.

The total attrition in the study may appear to be rather high. First of all the participation rates in this kind of population surveys are slowly decreasing in Sweden as in other European countries. It is nowadays almost impossible to attain more than 60% participation in questionnaire based population surveys, and the SLOSH study should therefore be regarded as successful. With regard to follow-up drop-out, it should be remembered that questions relating to leadership were mostly impossible to answer for those who were managers themselves and for self-employed participants. The 2006–2008 prospective analyses were based upon 3269–3730 out of 5,141, i.e. 64–73% of those who participated in 2006. 408 of the non-participants in this analysis (8% of the participants in 2006) were participants who had stopped working (mostly because they had reached normal retirement age) and had therefore not answered the questionnaire for working subjects. In addition there were in 2006 155 subjects (3% of the 5,141 participants in 2006) who were either managers (n = 581) or self-employed subjects (n = 299) who felt that they could not respond to the questions about their manager. This number increased among those who worked both in 2006 and 2008 (n = 3,644) to 8.5% in 2008; of those 3,644 participants 662 subjects were either managers themselves (n = 427) or self-employed (n = 235) and many of those (n = 309) therefore did not respond to the questions about managers. Among those who were working in both 2006 and 2010 (n = 2,998), 479 indicated that they had a managerial position with responsibility for subordinates and 179 answered that they were self-employed. In these two groups 265 felt that they were unable to answer questions about their manager, which results in an attrition of 8.8%. Accordingly from the 5,141 subjects in 2006 the main part of the loss to follow-up has natural reasons. Comparisons between the original sample and those who responded to SLOSH showed that women, older subjects (aged 50+) and married/cohabiting subjects as well as men and women with high education were overrepresented among responders. How these differences influence our findings is not clear. While depressive symptoms decrease with age in this sample no such relationship is observed for emotional exhaustion. Women report both more emotional exhaustion and more depressive symptoms than men [21]. Married and cohabiting subjects have been shown in other studies [22] to report less emotional exhaustion and depressive symptoms than single men and women. Accordingly the selection effect in our study causes mixed effects on the estimation of the true population scores of depressive symptoms and of emotional exhaustion.

A problem with the income variable is that part time work could introduce error. Parttime work is relatively common in Sweden among women. We therefore tested the regression models with this additional potential confounder. The results were very marginally affected and no conclusions were changed. We therefore chose not to include these calculations.

Low income and low education were related to higher scores for depressive symptoms in the present study. This may seem paradoxical since both these factors are indicators of low social class, and have been consistently related to elevated risk of developing depression in numerous studies [23]. In Scandinavia the relationship between income and depression and other psychiatric states has been the expected one in several studies [24], [25], [26] whereas the relationship between education and depression has been less consistent. Two Scandinavian studies performed by other epidemiological groups [24], [25] showed no significant relationship whereas a Danish study did show the expected relationship between low education and high prevalence of depression [26]. These inconsistencies could be due to a number of factors. The outcome factor depression has been assessed by means of a standardised clinical interview in one of the studies [26] whereas standardised self report questionnaires were used in the other studies as well as in our own study. Participants with high education may have a stronger tendency to overreport psychological symptoms than participants with low education and vice versa.

The fact that the “non-listening” score showed stronger relationships with social variables and also exhibited larger variations over study years than the “self centered” leadership factor could mean that the extent to which management listens to employees depends more on social interactions and that it may therefore be more sensitive to general changes in financial and social climate in a society. When the composite “self-centered” leadership variable was divided into its three items which were analysed with regard to variation over time, the most interactive item “non-participating” did indeed show variation over time in the same way as “non-listening”. The two other items did not show any variation over time. Perhaps the “self centered” leadership pattern could be regarded more as an individual characteristic than an interactive phenomenon. There was no relationship at all between income and “self centered” leadership score.

The prospective predictions using leadership scores were more successful for depressive symptoms than for emotional exhaustion. For the predictions of depressive symptoms it became clear that the introduction of the two work environment variables demands and decision latitude reduced the predictive value of the “non-listening” leadership score, which is an indication that part of the relationship between leadership score and employee health is due to this type of leader influence on the work environment. This partly supports the result of a Danish longitudinal study, showing that there was no direct relationship between transformational leadership and employee well-being, but a significant indirect relationship that was mediated through work environment factors [19].

The employees' perception of their manager contributed partly to the two-year predictions of their own mental health, particularly depressive feelings. In particular the observed effects of “non-listening” leadership were “mediated” statistically by the manager's influence on the psychosocial work environment (in particular psychological demands).

Our findings provide additional support to previous research emphasizing the importance of educational level - which was important in predictions particularly of emotional exhaustion. It was observed that high education level was related to higher scores of emotional exhaustion. This finding is in line with other research [11].

The improvement in psychosocial working conditions and leadership is associated with subsequent improvement in employee health has been shown recently by another Swedish group [20].

Our findings illustrate the importance of socio-economic factors to perceived management in the work place in Sweden during the later part of the 2000:s. The mechanisms behind the weak but consistent and significant relationship between low education level and “non-listening” leadership are unknown. Explanations may be related either to established communication patterns that are different in different socio-economic groups or to perceived differences in power sharing. Interpretations of the directions of the relationships should always be made with due caution in epidemiological studies. Accordingly our data cannot disentangle to what extent the relationships between leadership and employee health that we have observed are mediated by the leader's influence on work environment factors or whether both the leader and the work environment are influenced by common organizational factors that are beneficial to the health of the employees.
